# Detection of occult atrial fibrillation with 24-hour ECG after cryptogenic acute stroke or transient ischaemic attack: A retrospective cross-sectional study in a primary care database in Israel

**DOI:** 10.1080/13814788.2021.1947237

**Published:** 2021-07-09

**Authors:** Ori Liran, Tamar Banon, Alon Grossman

**Affiliations:** a Maccabi Healthcare Services, Tel-Aviv, Israel; b Maccabitech Institute of Research and Innovation, Maccabi Healthcare Services, Tel Aviv, Israel; c Department of Internal Medicine B, Rabin Medical Center, Petah Tikva, Israel

**Keywords:** Atrial fibrillation, 24-hour Holter ECG, ischaemic stroke, transient ischaemic attack, primary care

## Abstract

**Background:**

Ischaemic stroke or cerebrovascular accident (CVA) due to occult atrial fibrillation (AF) may cause severe morbidity and mortality. Diagnosing occult AF can be challenging and there is no consensus regarding the optimal duration of screening. A 24-hour Holter electrocardiogram (ECG) is frequently employed to detect occult AF following ischaemic CVA.

**Objectives:**

Demonstration of occult AF detection rate using a 24-hour Holter ECG in a primary care setting with descriptive analyses of independent variables to compare AF detected and non-detected patients.

**Methods:**

This retrospective cross-sectional study utilised primary care data and included patients 50 years and older with a new CVA or transient ischaemic attack (TIA) diagnosis followed by a 24-hour Holter examination within 6 months, between 01 January 2013 and 01 June 2019. The analyses included descriptive statistics comparing demographics and clinical characteristics in patients who had AF or Atrial Flutter (AFL) detection to those who did not.

**Results:**

Out of 5015 eligible patients, 66 (1.3%) were diagnosed with AF/AFL, with a number needed to screen of 88.5. Compared with those without AF/AFL detection, those diagnosed were older (75.42 ± 7.89 vs. 69.89 ± 9.88, *p* = 0.050), had a higher prevalence of hypertension (80.3% vs. 66.8%, *p* = 0.021) and chronic kidney disease (CKD) (71.2% vs. 44.2%, *p* < 0.001).

**Conclusion:**

24-hour Holter has a low AF/AFL detection rate. Older persons and those with hypertension or CKD are more likely to be detected with AF/AFL using this method.

KEY MESSAGES

- A 24-hour Holter electrocardiogram (ECG) is frequently employed to detect occult AF following ischaemic CVA or TIA.
- Real-world data demonstrates detection rates of 1.3% using this method.
- 24-hour Holter monitoring serves as an initial screening tool, yet a more efficient method for prolonged monitoring should be applied.

## Introduction

Ischaemic stroke due to occult atrial fibrillation (AF) may cause severe morbidity and mortality [1,2]. Diagnosing occult AF can be challenging due to its asymptomatic and intermittent nature [3,4] and requires electrocardiogram (ECG) monitoring. Several studies have shown that a longer duration of monitoring leads to higher AF detection rates [5–10]. One of these studies, performed by Gladstone et al., demonstrated a 3.2% detection rate using a 24-hour Holter ECG compared to a 16.1% detection rate using 30-day Holter monitoring [6]. There is no consensus regarding the optimal duration of screening for occult AF. The American Heart Association recommends prolonged (30 days) rhythm monitoring for patients who have experienced an acute stroke or transient ischaemic attack (TIA) within 6 months of the index event [11]. In contrast, The European Society of Cardiology recommends a short-term ECG followed by continuous ECG monitoring for at least 72 h (class I grade B recommendation), and long-term non-invasive ECG monitors or implanted loop recorders (class II grade B recommendation) should be considered [12]. In comparison to the American and European guidelines, current Israeli guidelines published in 2009 recommend ECG monitoring for 24–48 h [13]. Despite low detection rates, the use of a 24-hour Holter ECG is a common practice among primary care physicians in Israel, but there is no real-world data regarding the detection rates of this practice in a primary care setting.

This study aims to demonstrate the real-world AF detection rate in a primary care setting for patients after a cerebrovascular accident (CVA) or TIA and compare clinical aspects between AF patients and patients without AF diagnosis.

## Methods

### Data source

The study used de-identified data from the Maccabi Healthcare Services (MHS) central computerised database. MHS is the second-largest state-mandated healthcare provider in Israel, serving more than 2.5 million members (over 25% of the national population) and is a representative sample of the Israeli population. This fully computerised database captures all information on patient interaction (including demographics, visits, diagnoses, imaging, medication prescriptions, medication dispenses, procedures and laboratory measurements). The MHS database contains clinical data and is continuously updated and monitored internally by the medical informatics department.

### Study population and design

This retrospective cross-sectional study included primary care patients 50 years and older with a new CVA or TIA diagnosis followed by a 24-hour Holter examination (index date) within the next 6 months, between 01 January 2013 and 01 June 2019. Patients included in this study were outpatients visiting an MHS clinic for the administration of a 24-hour Holter ECG post CVA or TIA diagnosis. All patients with haemorrhagic stroke or other known reasons for stroke such as disseminated intravascular coagulopathy syndrome, subdural haemorrhage, brain aneurysm and subarachnoid haemorrhage, and patients with prior AF, Atrial flutter (AFL) or antiphospholipid syndrome (APS) diagnoses were excluded from the study. The *International Classification of Diseases version 9* (ICD-9) codes were used for data extraction and to identify patients with CVA, TIA, or APS. The 24-hour Holter examination results were automatically recorded and analysed, followed by a manual revision by an MHS cardiologist.

Furthermore, patients who were not MHS members for at least 12 months before the 24-hour Holter examination or those who left MHS during the study period were excluded.

To ensure a complete and clear report, the ‘Strengthening the Reporting of Observational Studies in Epidemiology’ (STROBE) checklist was applied as an assessment tool for methodological quality [14]. The STROBE checklist was used to guide for adequately reporting this research study.

### Variables

All variables describing chronic diseases, such as diabetes, coronary heart disease (CHD), cancer, hypertension, osteoporosis, chronic obstructive pulmonary disease (COPD), chronic kidney disease (CKD), asthma, and dyslipidemia were extracted from the patient records in the MHS data registries or from medical records that included a disease diagnosis code and diagnosis date. Chronic diseases were measured at Holter examination date and those who had any prior record were categorised as ‘ever’ since 1998.

Age was calculated from the 24-hour Holter date and smoking was divided into ‘ever’ and ‘never’ categories, defining patients who were considered smokers at least once in their medical visits or those who were never considered smokers. Body Mass Index (BMI) was calculated by patients’ records of height and weight; patients were categorised as either underweight (BMI < 18.5), normal weight (BMI = 18.5–25), overweight (BMI = 25–30), or obese (BMI > 30).

Smoking and BMI variables were measurements taken during physician examinations or hospital visits, therefore, data were not recorded for all patients and was categorised as ‘missing’. These measurements were taken within 12 months prior to the Holter examination date.

### Statistical analyses

The analyses included descriptive statistics comparing adult patients, ages 50 years and older, who had AF or AFL detection to those who did not. Patient characteristics such as age, sex, and other comorbidities were presented and compared. To evaluate the statistical significance between the two groups, a Student’s *t*-test was used for normally distributed continuous variables, such as age and days to Holter administration, and a Pearson’s Chi-squared test was used for categorical variables.

The ‘missing’ values were included as a category for Smoking and BMI, where Chi-square analyses were also applied. Less than 1% of the cohort had ‘missing’ data in both variables, likely due to recorded measurements during patients’ recent hospitalisations or clinician visits for CVA/TIA or Holter administration.

### Ethics

Approval was obtained from the Institutional Review Board (IRB) and Ethics Committee of MHS for the purposes of accessing and analysing the data. Individual patient informed consent was not required because of the anonymized nature of the patient records.

## Results

A total of 5,102 patients in MHS had records of CVA or TIA with a 24-hour Holter ECG in the following 6 months. However, 56 patients were excluded due to a record of APS and 31 patients were excluded due to leaving the MHS healthcare system or having less than 12 months of prior registration. Thus, 5015 patients were eligible for this study.

Sixty-six of these patients (1.3%) were diagnosed with AF or AFL, with a number needed to screen of 88.5. Patient demographics and comorbidities, presented in Table 1, highlight certain statistical differences. Notably, those diagnosed with AF/AFL were older (75.42 ± 7.89 years vs. 69.89 ± 9.88 years; *p* = 0.05) and had a higher prevalence of hypertension (80.3% vs. 66.8%; *p* = 0.02) and chronic kidney disease (71.2% vs. 44.2%; *p* < 0.001). The mean number of days from CVA or TIA diagnosis to 24-hour Holter exam was 63.85 days in the AF/AFL group and 51.46 days in patients who were not diagnosed with AF/AFL (*p* = 0.044).

**Table 1.** Comparison between 24-hour Holter patients with no AF/AFL detection vs. patients with AF/AFL detection up to 6 months after a CVA or a TIA (*n* = 5015).

	No AF/AFL detection	AF/AFL	*p*-Value
Number of patients (%)	4949 (98.7)	66 (1.3)	
Mean age (±SD)	69.89 (9.88)	75.42 (7.89)	0.050*
Average number of days to Holter exam (±SD)	51.46 (49.38)	63.85 (59.15)	0.044*
Sex			
Number of males (%)	2636 (53.3)	31 (47.0)	0.309
Smoking			
Never – number (%)	4258 (86.0)	60 (90.9)	0.505
Ever – number (%)	678 (13.7)	6 (9.1)	
Missing – number (%)	13 (0.3)	0	
Number of patients with diabetes (%)	1561 (31.5)	27 (40.9)	0.104
Number of patients with CHD (%)	2106 (42.6)	34 (51.5)	0.144
Number of patients with cancer (%)	908 (18.3)	13 (19.7)	0.778
Number of patients with hypertension (%)	3307 (66.8)	53 (80.3)	0.021*
Number of patients with osteoporosis (%)	1326 (26.8)	23 (34.8)	0.143
Number of patients with COPD (%)	403 (8.1)	4 (6.1)	0.538
Number of patients with CKD (%)	2185 (44.2)	47 (71.2)	<0.001*
Number of patients with Asthma (%)	795 (16.1)	8 (12.1)	0.386
Number of patients with dyslipidemia (%)	2685 (54.3)	34 (51.5)	0.657
BMI			
Underweight – number (%)	38 (0.8)	0	0.747
Normal Weight – number (%)	1265 (25.6)	15 (22.7)	
Overweight – number (%)	2106 (42.6)	26 (39.4)	
Obesity – number (%)	1534 (31.0)	25 (37.9)	
Missing – number (%)	6 (0.1)	0	

*Statistical significance.

AF: atrial fibrillation, AFL: Atrial flutter; CVA: cerebrovascular accident; TIA: transient ischaemic accident; SD: standard deviation; CHD: coronary heart disease; COPD: chronic obstructive pulmonary disease; CKD: chronic kidney disease; BMI: body mass index.

## Discussion

### Main findings

Among 5,015 patients in MHS with records of CVA or TIA followed by a 24-hour Holter ECG, we detected 66 patients (1.3%) with AF/AFL. Those detected were older (75.4 vs. 69.9) and more often suffered from hypertension (80.3% vs. 66.8%) and CKD (71.2% vs. 44.2%) as compared to patients in whom no AF/AFL was detected.

### Interpretation

Prior small-scale studies showed similar results, suggesting that this method has a low detection rate. Doliwa et al., showed a detection rate of 2.0% using 24-hour Holter, in a study including 249 patients [15]. Two additional studies performed by Shibazaki et al. and Schuchert et al. demonstrated a detection rate of 2.2% and 1.2%, respectively [16,17]. The latter was an outpatient setting study, where this current study supports these findings in a first large-scale population-based research.

In contrast, results are conflicted with a study performed by Alhadramy et al. with a detection rate of 9.2% [18]. Their study was stroke unit-based and included 426 patients as opposed to the primary care-based population in this study. Another stroke unit research study using a 24-hour Holter upon admission, due to a CVA or a TIA, showed a much higher detection rate [19], suggesting that this method might be applicable in the immediate setting of stroke care.

Furthermore, the results from this study showed significant statistical differences between the patients who were diagnosed with AF and the patients who did not receive such diagnosis – specifically older age, hypertension and CKD. These are all known risk factors for AF/AFL and are supported by previous studies [20–22], suggesting a higher detection rate in these patients than younger patients without hypertension or CKD. Thus, older patients, particularly those with hypertension and CKD are at a higher risk for AF/AFL as caused by CVA and may require a more aggressive approach for the detection of AF/AFL, including more prolonged periods of monitoring.

This study demonstrates a statistically significant difference in the mean days between CVA or TIA diagnosis and 24-hour Holter examination (63.85 vs. 51.46 days) compared to patients without AF/AFL detection. A study performed by Sanna et al., using an insertable cardiac monitor demonstrated a median of 84 days from randomisation to AF detection [23]. Newly published studies used implantable loop recorders and resulted in an average time to first AF episode of 108 days [24] and 121 days [25]. Overall, these findings are consistent with our results and suggest that performing an outpatient delayed cardiac monitoring with relation to the stroke did not lower the detection rates.

Recent studies propose that the implementation of loop recorders should be used to detect occult AF, where their results demonstrate high detection rates [8,22], however, this method may not be feasible in the primary care setting. Therefore, the need for a cost-convenient and effective detection method has led to the research of smartwatches and photo-plethysmograph wrist-watch sensors as an alternative for loop recorders [26–28]. A meta-analysis performed by Afzal et al., for AF detection after cryptogenic stroke resulted in a detection rate of 13.3% using wearable devices with a median duration of 21 days [8]. Current Israeli guidelines were written in 2009 and predate more advanced and feasible technologies for prolonged monitoring in the outpatient setting. The accumulating evidence on this topic is sufficient to recommend prolonged monitoring periods for post-stroke patients. Yet, the high cost of prolonged monitoring is certainly a limit to the universal implementation of this recommendation. We believe that our results provide additional information that may eventually influence changes in the current Israeli guidelines for outpatient monitoring.

Additional AF screening studies in primary care found higher rates of AF detection [29,30]. This difference may be due to the exclusions applied in this study, as patients with a previous diagnosis of AF were excluded from the analysis. As AF is ubiquitous in stroke patients, one would expect most cases to have been diagnosed prior to or during the hospitalisation due to CVA, but our results suggest that active surveillance for this arrhythmia is still required.

### Strengths and limitations

This study’s main limitation is the data collection and interpretation method for AF and AFL diagnosis, which was based on the free text typed by physicians. Thus, if there were any mistakes in the free text or if physicians failed to enter their notes correctly, MHS would have no record in the database. It is possible that some patients who had AF or AFL within the 7-year study period were excluded from this research due to this limitation in the data. On the contrary, using real-world data permitted this study’s population to consist of over 5000 patients with the specific inclusion criteria in a specific study period over several years.

### Implications for practice

This study’s results suggest that the 24-hour ECG is not sufficient to detect AF or AFL in a primary care setting, as the rate of detection is significantly lower than the rate reported for more extended monitoring periods. A 24-hr monitoring period has a low sensitivity, which is why current European and American guidelines recommend longer monitoring periods [11,12]. Patients could be provided with auto-triggered monitors and smartwatches as a preferred alternative for the 24-hour ECG. Yet, due to its non-invasive nature and low cost, the 24-hour ECG is recommended as an appropriate method for initial AF or AFL screening following CVA/TIA. However, if the 24-hour ECG monitoring does not detect this arrhythmia, particularly in high-risk patients (those with hypertension or CKD), more prolonged monitoring would be appropriate.

## Conclusion

In conclusion, the 24-hour ECG Holter has a low AF/AFL detection rate and certain high-risk patients, namely older persons and those with CKD and hypertension, are more likely to be diagnosed with AF/AFL using this method.
